# Removal of Mn (II) by Sodium Alginate/Graphene Oxide Composite Double-Network Hydrogel Beads from Aqueous Solutions

**DOI:** 10.1038/s41598-018-29133-y

**Published:** 2018-07-16

**Authors:** Xiuzhen Yang, Tengzhi Zhou, Bozhi Ren, Andrew Hursthouse, Yuezhou Zhang

**Affiliations:** 1 College of Civil Engineering, Hunan University of Science and Technology, Xiangtan, 411201 China; 2 School of Science & Sport, University of the West of Scotland, Paisley, PA1 2BE UK; 3 Pharmaceutical Sciences Laboratory, Faculty of Sciences and Engineering, Åbo Akademi University, FI-20520 Turku, Finland

## Abstract

After the successful preparation of empirical double network hydrogel beads from graphene oxide/sodium alginate(GO/SA), its cationic metal adsorption performance in aqueous solutions were investigated. Taking Mn(II) as an example, the contribution of several factors including pH, bead dosage, temperature, contact time and initial concentration ions to adsorption efficiency were examined. The Transmission Electron Microscopy (TEM) results indicate that the GO/SA double (GAD) network hydrogel bead strongly interpenetrate and the adsorption of Mn(II) is mainly influenced by solution pH, bead dose and temperature. The GAD beads exhibit an excellent adsorption capacity of 56.49 mg g^−1^. The adsorption process fit both *Pseudo*-second order kinetic model (R^2^ > 0.97) and the Freundlich adsorption isotherm (R^2^ > 0.99) and is spontaneous. After seven rounds of adsorption-desorption cycle, the adsorption capacity of GAD hydrogel remained unchanged at 18.11 mg/g.

## Introduction

Due to the weathering and erosion of geological environment and the excessive discharge of wastewater from mining, smelting and other industrial sectors^1^, the issue of heavy metal pollution transferring to waters remains and will continue to be a worldwide challenge^2–4^. Contamination of heavy metal to the water environment is delivered through the food chain and endangers human health in the end^5,6^ and widely recognized as a global issue^7^. Manganese is extensively used in metallurgy, electronics, battery manufacture, chemical industry as well as an important element in production of alloys^8^. Although it can exist in multiple oxidation states (Mn^+2^, Mn^+3^, Mn^+4^, Mn^+6^ and Mn^+7^), the divalent form Mn^2+^ predominates in most contaminated waters^9^. The existence of Mn^2+^ can lead to odour, chromaticity in water and harm to the human central nervous system and endocrine system when more than 10 mg intake per day^10–12^. Some of mining areas are seriously suffering from Mn^2+^ pollution and the pollution treatment lag far behind the desirability, such as Xiangtan city of Hunan province, China. Therefore, ones have to confront this challenge.

Many attempts to deal with heavy metal pollution have been reported, such as chemical precipitation, ion exchange, adsorption, bioremediation and electrochemical treatment^13–15^. Among them, adsorption is of interest given its advantages of cost effectiveness, high efficiency and less secondary pollution^16–18^. The component of adsorbents is varied^19,20^, including clays, solid industrial wastes, organic polymer materials but many of them have low adsorption capacity and is difficult to recycle^21^.

Graphene oxide (GO) has received a lot of attentions as adsorbents for contaminant removal from water. Compared to other adsorbents, graphene oxide adsorbents possess super high specific surface area (2630 m^2^/g) and many chemical functionalities such as hydroxyl, epoxy, and carboxyl groups, facilitating GO to be further hybridized with other materials to form composite materials^22^. The early stage research focused on the π-π stacking interaction and the Van der Waals force between graphene oxide layers, which compromised its adsorption capacity to contaminants^23^.

Alginate, an ionic polysaccharide mainly refined from the cell walls of brown algae, is linear arranged by homopolymeric blocks of (1–4)-linked β-D-mannuronate (M) and its C-5 epimer α-L-guluronate (G) residues, making it hydrophilic, biocompatible, nontoxic, and editable^24,25^. Chelation of divalent cations with G block of alginate to generate hydrogels is widely observed and is one of main forms alginate-based hydrogels. These alginate hydrogels were usually used as biomedical application and its usage in wound healing, drug delivery, and tissue engineering has been detailed reviewed^26^. Other applications, such as heavy metal depletion^27^ was also exploited. Notwithstanding, the lack of mechanical stability and the ability to tune it often compromise its broader applications because of ion exchange between divalent ions in the alginate and monovalent ions in solutions. The stability of alginate hydrogel was improved by hybridization of linear polyethyleneimine and Ca^2+^ as polyelectrolytes, enabling the hydrogel stiff enough to function as cell scaffold material^28^. Recently, Yuan *et al*.^27^ introduced graphene into alginate hydrogel to enhance mechanical strength from 0.29 MPa (pure alginate hydrogel) to 2.14 MPa (GAD-network), and increase adsorption capacity of Cu^2+^ and Cr_2_O_7_^2−^ up to 169.5 mg g^−1^ and 72.46 mg g^−1^ individually. Not only cationic metal adsorption, small organic compounds can also bind to graphene/alginate hydrogel, such as ciprofloxacin^29^. Moreover, this hydrogel was further modified into porous skeleton by using CaCO_3_ as pore formation agent therefore achieved remarkable enhancement of adsorption removal of ciprofloxacin from aqueous solutions^30^. Notwithstanding, another hurdle for alginate hydrogel use in practice is reusability. The normal hydrogel will swell in solution. If this hydration and volume expansion are inevitable and irreversible, the mechanical properties will be badly deteriorated. One of the solution is to build up double-network (DN) hydrogel^31^ since it has been demonstrated that DN represents higher specific surface area, better thermal stability than single network and more resistant to ionic strength^32^: typical double-network gels consist of two intertangling polymeric components with complementary structural and mechanical properties. One component is stiff and serve as the skeleton while the another one remains elastic and loosely networks the hydrogel. In the end elastomers with reinforced in stiffness and toughness are obtained^33–36^.

Nowadays topological, nanocomposite and DN hydrogels have become of interest because of their excellent mechanical performance^31–33^. Typical DN hydrogel comprises of two different network structures, one is polyelectrolyte network with high density of cross-linking, and another with low cross-linking or non-crosslinking neutral network structure^37^. Due to the 3-dimensional nature of the GO and chemical modifiability, it can effectively integrate with alginate to form a DN nanohydrogel.

In this experiment, a new DN hydrogel bead comprising GO and sodium alginate (SA) (GO/SA) bead was prepared. This new GAD hydrogel has potential as an adsorbent because of the following advantages: (1) the hydrogel bead integrates the characteristics of high specific surface area and thermal stability of graphene oxide and the biocompatibility of sodium alginate; (2) the hydrogel bead retains the functionality of GO and allows reaction in aqueous systems with improved biocompatibility; (3) the hydrogel bead can be quickly separated from wastewater, recycled and regenerated for sustained application. We describe here the performance characteristics of GAD hydrogel beads in the treatment of Mn(II) contamination.

## Materials and Methods

### Reagents and Instruments

Graphene oxide prepared by modified Hummers’ method^38^. Due to the divalent form of manganese predominates in most waters^9^, the manganese test solution prepared by dissolving manganese chloride tetrahydrate (MnCl_2_·4H_2_O, AR, Tianjin Kemiou Chemical Reagent Co., Ltd,. China) in deionezid water, and pH adjusted using hydrochloric acid and sodium hydroxide in all experiment. The sodium alginate was chemically pure and purchased from Sinopharm Chemical Reagent Co,. Ltd,. China. Anhydrous calcium chloride (CaCl_2_) was analytically pure and afforded by Tianjin Fengchuan Chemical Reagent Technologies Co., Ltd,. China. Ascorbic acid was analytically pure and provided by Xilong Sicentific Co., Ltd., china.

The specific surface area, total pore volume and pore size distribution were identified by nitrogen adsorption/desorption at 77.4 K using ASAP 2020(Micromeritics, USA), and all the beads were degassed at 373 K before the measurements. The thermo gravimetric analysis(TGA) was measured under a nitrogen atmosphere on Netzsch STA449F3(Germany) in the range temperature from room temperature to 650 °C with calefactive rate of 10 °C/min. Transmission electron microscopy (TEM) images were captured by JEOL JEM-2100 TEM (USA) at 200 kV. Scanning electron microscope (SEM) images were captured by JSM-6390LV (Japan) coupled with an energy-disperse X-ray analyzer (EDX). Fourier-transform-infrared spectroscopy (FT-IR) spectra were recorded on a Perkin Elmer Spectrum 100 spectrometer(USA). Atomic absorption spectra were acquired by atomic absorption spectrophotometer AA-7003 (East & West Analytical Instruments, Inc., China) to analyze Mn(II). The adsorption study was performed in water-bathing constant temperature vibrator THZ-82 (Jintan Ronghua Instrument Manufacture Co., Ltd, Jiangsu, China).

### Preparation of GAD Hydrogel Beads

Sodium alginate was added into the water with magnetic stirring for 1 h. Then, the solution of sodium alginate was dripped into a 5 wt % solution of CaCl_2_ at neutral condition where the calcium ions and sodium alginate forms hydrogel beads, and sodium alginate beads(SA) were prepared.

Graphene oxide (20 mg) was dispersed into deionized water (50 ml) in an ultrasonic bath. Sodium alginate was added into the solution of graphene oxide and mixed with magnetic stirring for 1 h. Then, the graphene oxide and sodium alginate mixture was dripped into a 5 wt % solution of CaCl_2_ at neutral condition where the calcium ions and sodium alginate forms hydrogel beads by cross linking, and the GO roughly account for 2 wt% in the prepared beads. The hydrogel beads are then immersed into ascorbic acid solution in thermostat water bath at 90 °C for 8 h. Then, the double network structural has been formed. Lastly, beads were washed with deionized water three times and dried in an oven at 90 °C for 12 h.

### Adsorption experiment

The dried GAD hydrogel beads were used in subsequent adsorption experiments, which mainly study on the adsorption performance of GAD hydrogel beads in different conditions, such as initial pH, dosage of adsorbent, contact time, temperature and the initial concentration of Mn(II). Demanded amount of beads were added into 100 ml polyethylene bottle containing 50 ml of Mn(II) solution that adjusted pH value by 0.1 M HCl and NaOH solutions, and reaction in incubator shaker at a fixed temperature (298 K) and rotation speed (150 rpm). The residual Mn(II) in solution were monitored by atomic absorption spectrophotometer after a period of contact time.

The adsorption kinetics experiment was carried out at pH 6. A portion of beads (specify the mass added) was added into the solution of Mn(II) and left to react in the incubator shaker. The adsorption capacity was determined from the residual Mn(II) concentration in solution was monitored every 30 minutes for up to 210 minutes and continuously followed every 60 minutes for up to 450 minutes. According to the actual wastewater release characteristics from source of pollutant, the adsorption isotherm experiment was carried out with a dose of 150 mg beads and the initial concentration of Mn(II) solution in the range 10–200 mg/L.

The dose-response experiment was conducted at pH 9. A 50 ml portion of Mn(II) solution with the initial concentration of 10 mg/L was added into five 100 ml polyethylene bottles respectively. In the dosage range of 10–500 mg, the adsorption reaction was carried out in incubator shaker (298 K) for 210 min with duplicate samples.

When investigate the adsorption kinetics of hydrogel, 200 ml of Mn(II) aqueous solutions with initial concentrations of 10 mg/L, 50 mg/L, 125 mg/L were prepared respectively and pH adjusted to 6. A 600 mg portion of adsorbent was added into this solution, the reaction was carried out at 298 K with the speed of 150 rpm and the residual concentrations of Mn(II) were monitored over time.

Reusability represents an important factor to evaluate the performance of adsorbents, and the adsorption-desorption reliability of GAD has been investigated in this paper. Reusability was evaluated by using 0.1 mol/L HCl as desorbing agent. The initial concentration of Mn(II) was 200 mg/L and 150 mg of dose. After the implement of adsorption process at pH 6, the Mn(II)-loaded GAD was predicated, treated with HCl for 3.5 h, washed with deionized water, and dried in an oven at 90 °C. Thereafter, the recovered adsorbent was used in the adsorption-desorption experiment seven cycles.

## Results and Discussion

### Characterization

#### The structure of GAD

**Figure 1:**
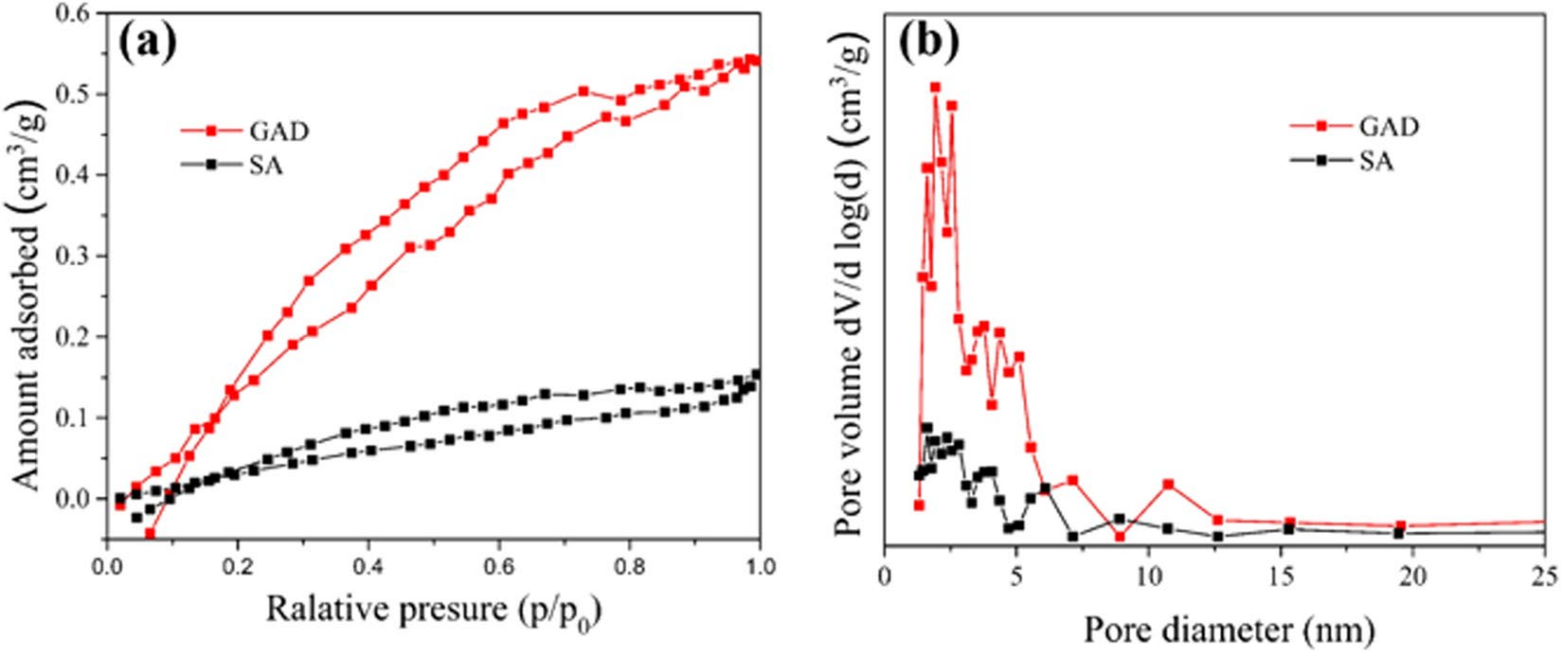
N_2_ adsorption and desorption isotherms (**a**) and pore size distributions (**b**) of GAD and SA.

The hydrogel beads prepared are spherical, with 1 mm diameter (Fig. S1). As showed in Fig. 1(a) and Table 1, the BET surface area of GAD is 2.6 m^2^/g, smaller than the recent report^39^. It is because the comparative lower GO content (only 2 w%) in GAD beads. However, approximately 8 times larger the surface area of GAD than SA beads is still achieved by integration small amount of GO in beads. The addition of GO led to the decreased average pore diameter from 2.8 to 1.3 nm, the increased total pore volume of GAD from 2 × 10^–3^ to 8 × 10^−3^ cm^3^/g and significant enhancement of the proportion of 1–5 nm sized pores which are the main contributor of the porosity of the GAD (Fig. 1(b)).” There is no direct evidence to show double network GAD was formed in this study, but DN should account for predominated population based on the hydrogel beads predation and proposed GAD composite assembly process, together with morphology comparison between typical single network of SA (Fig. S2a) and SA/GO composite.

**Table 1:** Physicalproperty of GAD and SA.

Characteristics	GAD	SA
Surface area (m^2^/g)	2.6	0.3
Average pore diameter (nm)	1.3	2.8
Total pore volume (cm^3^/g)	0.0008	0.0002

#### Thermal stability of GAD

**Figure 2:**
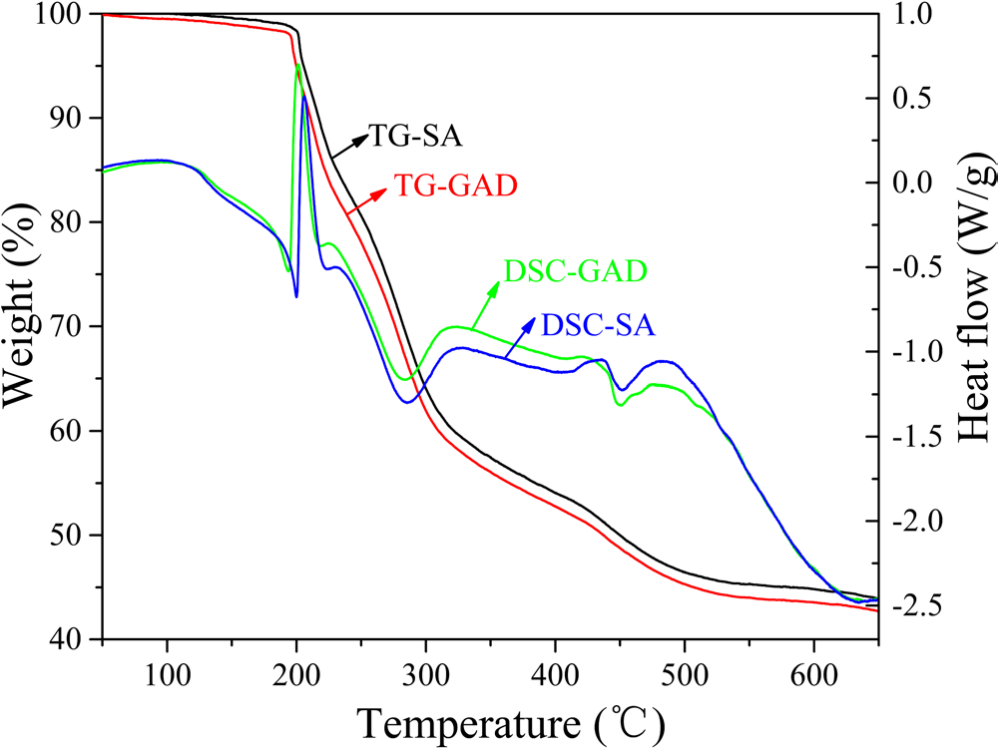
DSC-TG record of GAD (7.2 mg) measured in nitrogen.

The thermal stability of GAD and SA beads was characterized by the thermogravimetric analysis (TG) and differential scanning calorimetry (DSC). Figure 2 presents the DSC-TG result of GAD sample (7.2 mg) tested in N_2_ protective atmosphere. When it is below 180 °C, the weight loss of GAD and SA is mainly due to the evaporation of moisture in beads. The second stage of the weight loss of GAD was in the range of 180–300 °C, corresponded to the decomposition of labile oxygen-containing functional groups^39^. In the case of SA, the starting point for the decomposition shifted to 185 °C, a bit higher temperatures than 180 °C for GAD. This result probably because the loading of GO in SA lead to enhanced the thermal conductivity of GAD. The mass loss of GA and GAD occurred in the range of 300–550 °C, presumably due to the combustion of GO and the decomposition of the carboxyl group; above 550 °C, GAD and SA reduced further in weight due to the bulk pyrolysis of the carbon skeleton^40^.

#### TEM

**Figure 3:**
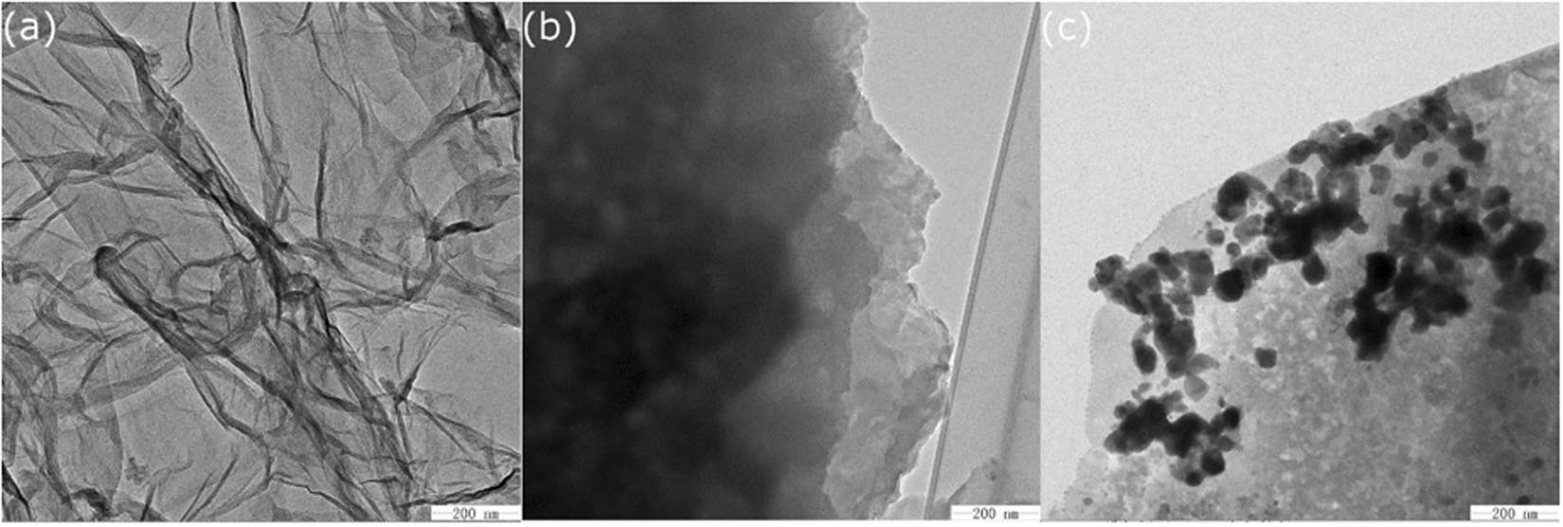
TEM of the (**a**) GO, the (**b**) GAD before adsorption and the (**c**) GAD after adsorption.

The TEM images, indicating intensity of electrons attenuated by the graphene oxide, GAD hydrogel beads platelets of different thickness showed wrinkled sheet like morphology with different transparencies (Fig. 3(a)). Smaller dark areas indicate the thick stacking nanostructure of several GO and/or graphene layers with some amount of oxidized functional groups, while higher transparency areas relate with much thinner films of few layers graphene oxide and resulting from stacking nanostructure exfoliation^41^. After ultrasonic dispersion, GO sheets will tend to separate from each other, implying significantly less surface area with low transparency of graphene layers might be observed according to previous study^42^. The sonication maximizes π-π stacking sites of graphene sheets, but the edge of the sheet remains partially superimposed each other^43^. As result, delaminated structure consisting of matrix composites stack can be observed from SEM images of GAD hydrogel, as shown in Fig. 1b. The irregular hackly protrusion from the margin may imply the hydrated state of hydrogel bead. The protrusion degree was marked by transparency contrast in different area. When cationic Mn(II) and hydrogel beads adsorption take place (Fig. 3(b)), large quantity of particles attaches on the surface. Although layered hydrogel bead structure was almost invisible, its’ edge become smoothened and pseudo-curved comparing to before adsorption, which may this indicate that the adsorbent effectively reacted with Mn(II) ions in solution.

#### FT-IR

**Figure 4:**
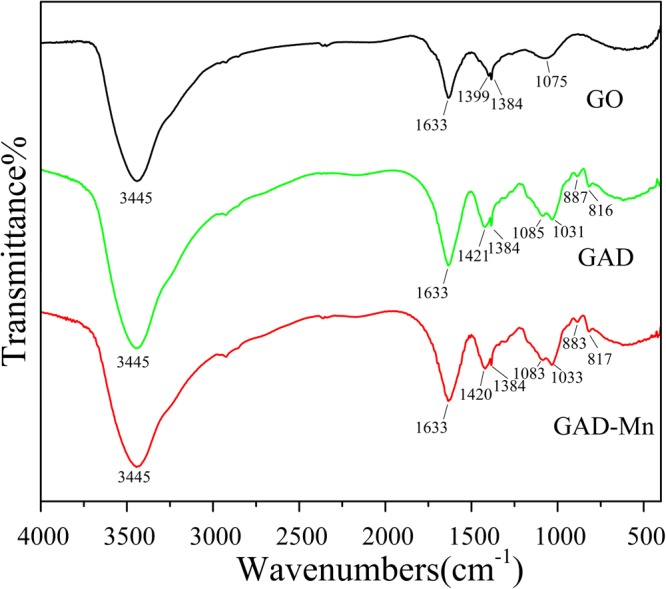
FTIR spectra of GO, GAD and GAD-Mn.

As shown in the Fig. 4, a strong stretching vibration appeared at 3445 cm^−1^, which is the characteristic absorption peak of -OH bond; the absorption peak at 1384 cm^−1^ and 1399 cm^−1^ signaled the bending vibration of -CH_3_; and the peak at 1633 cm^−1^ was induced by the vibration of C=C. Compared to the spectra of GO with GAD, four new bands appear at 1085 cm^−1^, 1031 cm^−1^, 887 cm^−1^, 816 cm^−1^ and the absorption peak at 1399 cm^−1^ blue shifts to 1421 cm^−1^, that indicated the graphene oxide fully reacted with sodium alginate in the preparation of the double network composite. When comparing GAD with GAD-Mn, no new characteristic absorption peaks are observed but three absorption peaks at 1420 cm^−1^, 1083 cm^−1^, 883 cm^−1^ remarks slight red shift occurrence. The absence of significant changes in IR spectra implies the adsorption process is physical mechanism oriented.

#### SEM-EDX

**Figure 5:**
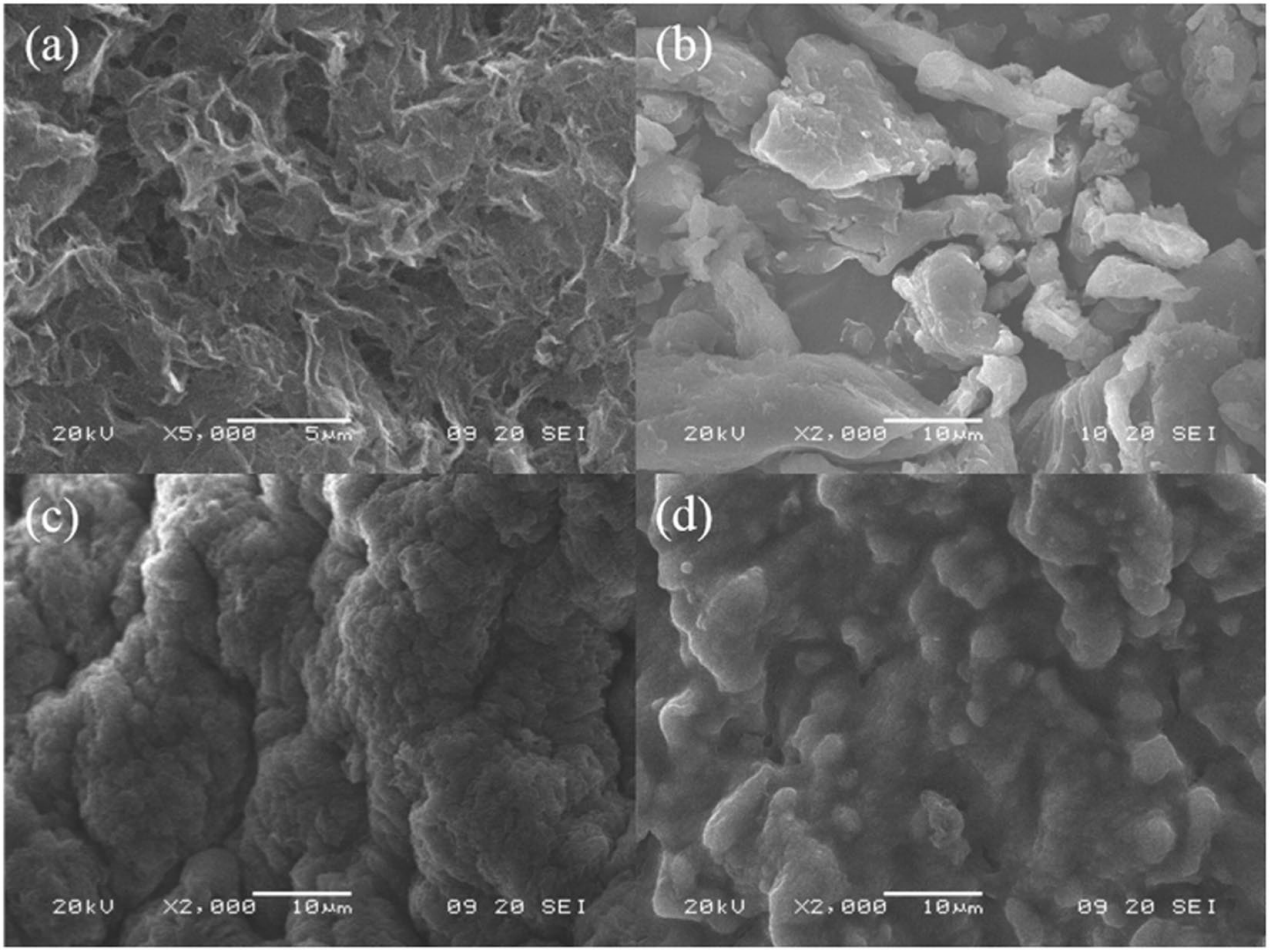
SEM of the (**a**) GO, the (**b**) sodium alginate, the (**c**) GAD before adsorption and the (**d**) GAD after adsorption.

**Figure 6:**
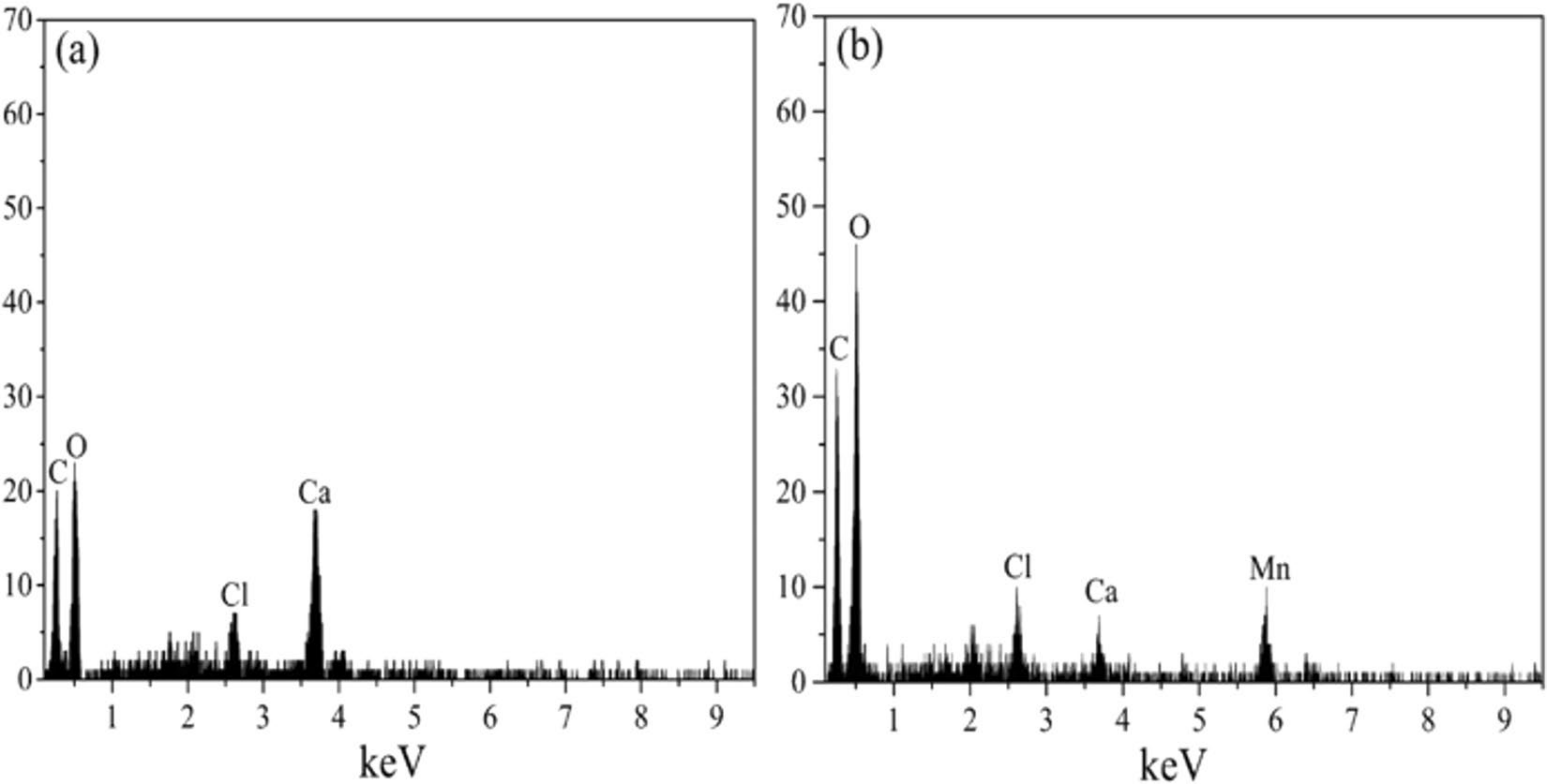
EDX of the GAD (**a**) before adsorption and the (**b**) after adsorption.

The SEM features of material in question are enumerated in Fig. 5, the surface of reduced GO is rough and pitted with craters. The layers of the GO are delaminated, forming a porous structure (Fig. 5(a)). While the surface of sodium alginate powder (Fig. 5(b)) is very smooth and not porous. After hydrogel preparation process, the GO and sodium alginate were fully reacted and formed the GAD beads. Plenty of wrinkle and pore on the surface of the fresh GAD beads (Fig. 5(c)), represented by peak and valley topography. On the contrary, such rise and fall landscape become blurred after Mn (II) ions adsorption (Fig. 5(d)), and many amorphous insoluble substances attached on the GAD beads surface that possibly compose of manganese. The adsorption of Mn (II) ions and successful incorporation of calcium ion into GAD hydrogel bead are cross-validated by energy dispersive X-ray analysis. In the preparation process, sodium alginate effectively cross linked with calcium ions in the CaCl_2_ solution (Fig. 6(a)), suggested by anionic chloride peak at 2.6 keV and 3.7 keV for cationic calcium. After adsorption, a new manganese characteristic peak appears at 5.8 keV (Fig. 6(b)), demonstrating the manganese ions were adsorbed by GAD beads.

### Batch Adsorption Study

The removal efficiency (%) of Mn(II) was calculated by the formula (1), and the adsorption capacity was calculated by the formula (2) in adsorption experiment.

1 $${ \% }\,{removal}=\frac{{C}_{0}-{C}_{e}}{{C}_{0}}\times 100$$

2 $${{\rm{q}}}_{{\rm{e}}}=\frac{({C}_{0}-{C}_{{\rm{e}}})\cdot V}{{\rm{m}}}$$

where the C_0_(mg/L) is the initial concentration of Mn(II) in solution; C_e_(mg/L) is Mn(II) concentration at equilibrium; V(L) is the volume of the solution and m(g) is the weight of adsorbent.

#### The Effect of pH

The Mn(II) in solution tends to precipitate at high pH values. It is also believed than electrostatic repulsion hinder the cationic metal ions to adsorb on the positively charged surface of GAD hydrogel at a low pH range of 2–3^44^. Therefore, the adsorption study was carried out in the pH range of 3 to 7, the initial concentration of manganese was 10 mg/L, the dosage of GAD was 3 mg/ml. The mixture was shaken at 298 K in an incubator shaker with the speed of 150 rpm for 210 min.

**Figure 7:**
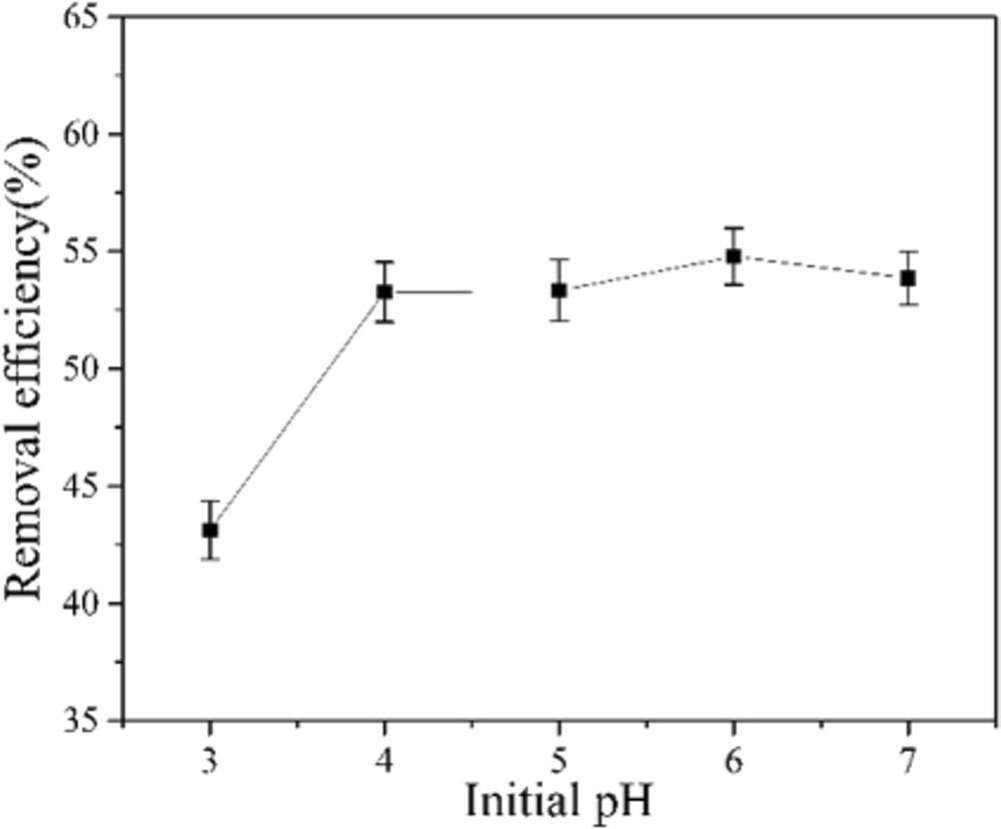
Effect of pH on adsorption efficiency of Mn(II).

Figure 7 showed the effect of pH on the adsorption performance of GAD hydrogel beads at the pH range of 3 to 7. In general, the removal efficiency of cationic Mn(II) is positively related with pH, but not linear. The adsorption of cationic manganese is hindered at low pH (e.g., 3.0 and 4.0). This is because H^+^ competition with manganese ions for adsorption sites in aqueous solution^19^. When the pH is above 4.0 until neutral 7.0, the adsorption efficiency of Mn(II) remains almost unchanged and reaches the adsorption balance points of 55%. The charges on the hydrogel beads surface are negative since the deprotonation reactions (i.e., –COOH–H + → –COO^−^) happen during the process. Hence the positive Mn(II) ions are readily adsorb on the negatively charged surface of GAD. However, there is no more deprotonated sites –COO^−^ are released with increasing pH, which may explain Mn(II) remove efficiency keep constant from mild acidic to neutral pH. When pH turns to basic, hydroxyl group on the surface of GO might get deprotonated alike^45^, therefore offer GAD hydrogel additional cationic ions adsorption ability. Therefore, increase Mn(II) remove efficiency was observed from under basic condition. The relative species of manganese are present in aqueous solutions in the forms of Mn^2+^, Mn(OH)^+^ and Mn(OH)_2_ at different pH. Mn^2+^ and Mn(OH)^+^ predominate the existence of manganese species at pH < 8.9^46^. Therefore, it is ionic interaction between cationic manganese species and deprotonated GAD drive the hydrogel absorption.

#### Effect of Sorbent Dose

**Figure 8:**
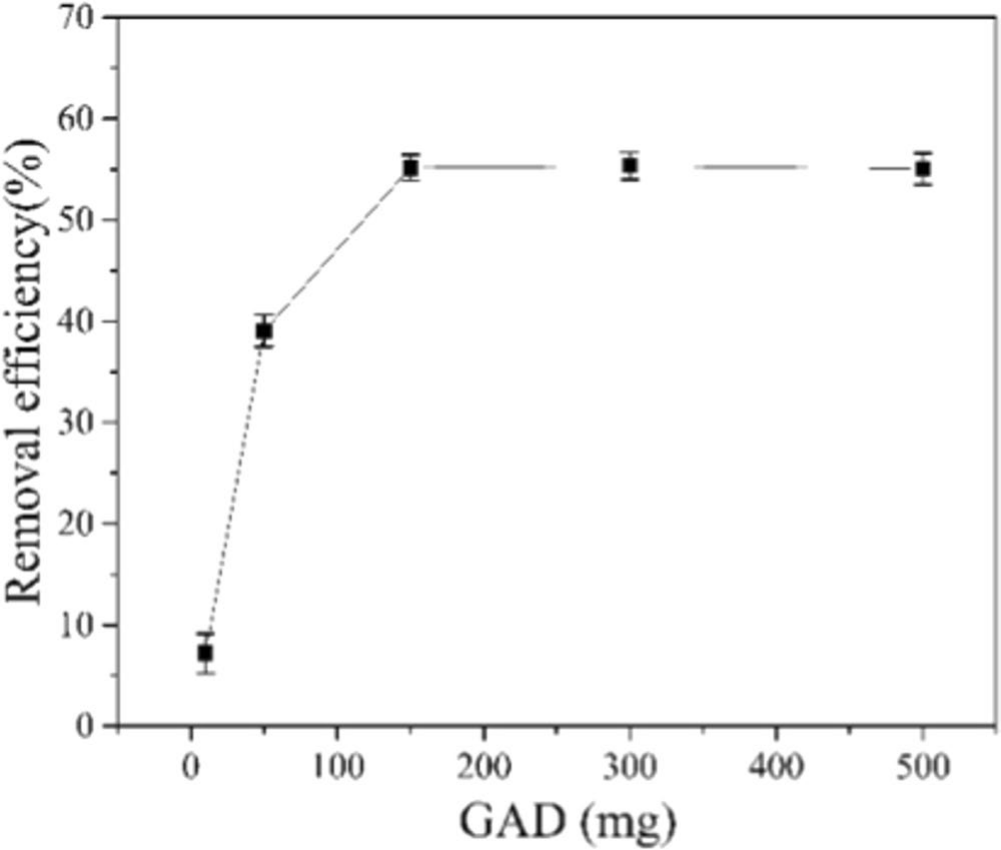
Effect of GAD dose on removal efficiency of Mn(II).

As is shown in the Fig. 8, the removal efficiency of Mn(II) was positively correlated with GAD hydrogel beads at the low dosage. The removal efficiency of Mn(II) ions in aqueous solution was 7% for a 10 mg dosage. With the increase of hydrogel beads from 10 to 150 mg, the removal efficiency is enhanced to 55%. This is an expected result because the more adsorbent offer more adsorption sites, which will favor the adsorption of Mn(II). The adsorption reaches equilibrium at a dose of 150 mg and overdose (above 150 to 500 mg) did not improve the Mn(II) clearance. With the amount of the adsorbent increases, the relative number of metal ions around adsorbent particles decreases or ratio of the number of the adsorbent to metal ions increases. As result, not all available adsorption sites are fully utilized, therefore the total Mn(II) removal remain constant, the similar result was observed in previous study^47^.

#### Adsorption Kinetics

The study on the kinetic characteristic of use GAD hydrogel beads to remove Mn(II) in aqueous solution is beneficial to the practical application of the adsorbents in wastewater treatment systems and to understand the adsorption mechanism^48^. The experimental data was tried to fit the models of *pseudo*-first order, *pseudo*-second order and intra-particle diffusion were applied to the kinetic data. Fitting formulas was shown below^49^:

3 $$\mathrm{ln}({q}_{e}-{q}_{t})=\,\mathrm{ln}\,{q}_{e}-{k}_{1}t$$

4 $$\frac{t}{{q}_{t}}=\frac{1}{{k}_{2}{q}_{e}^{2}}+\frac{1}{{q}_{e}}t$$

5 $${q}_{t}={k}_{i}{t}^{1/2}+a$$

where *q*_e_(mg/g) is the adsorbent Mn(II) quantity at equilibrium; *q*_t_(mg/g) is the adsorbent quantity at time t; *k*_1_ and *k*_2_ are the first and second order equilibrium rate constants, respectively. *k*_i_ is the adsorption efficiency constant of the intra-particle diffusion model.

**Figure 9:**
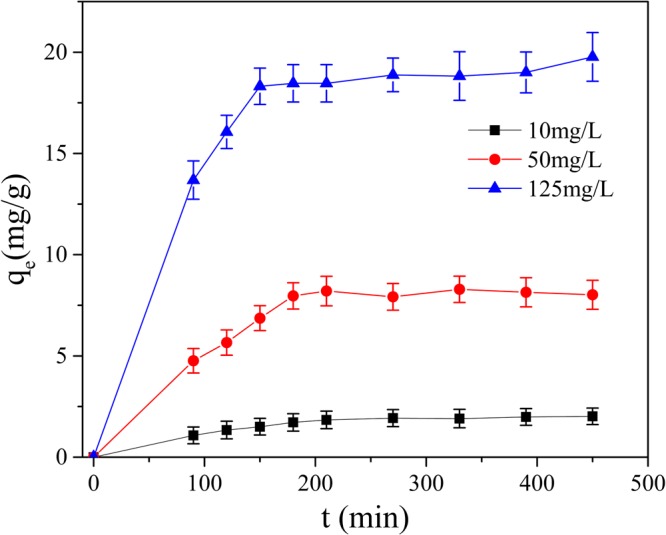
Effect of contact time on removal Mn(II) by GAD.

**Figure 10:**
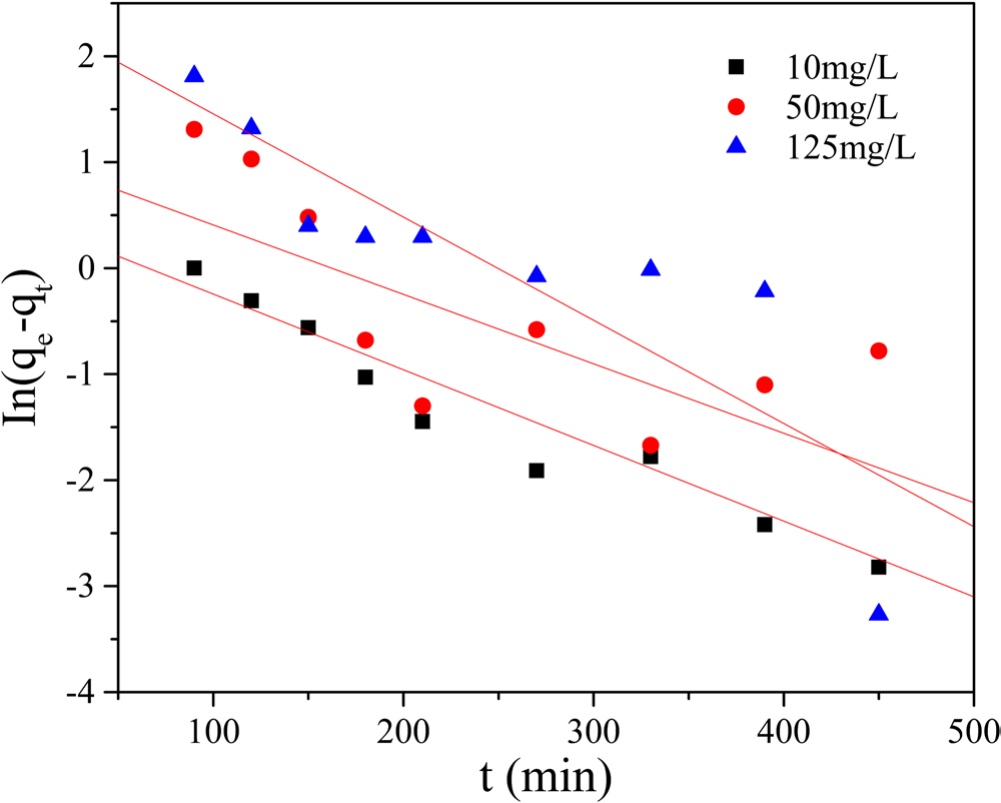
*Pseudo*-first order reaction model.

**Figure 11:**
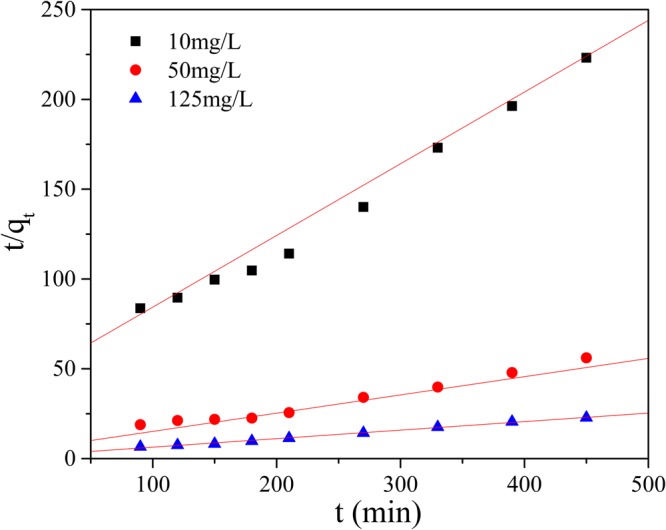
*Pseudo*-second order reaction model.

**Figure 12:**
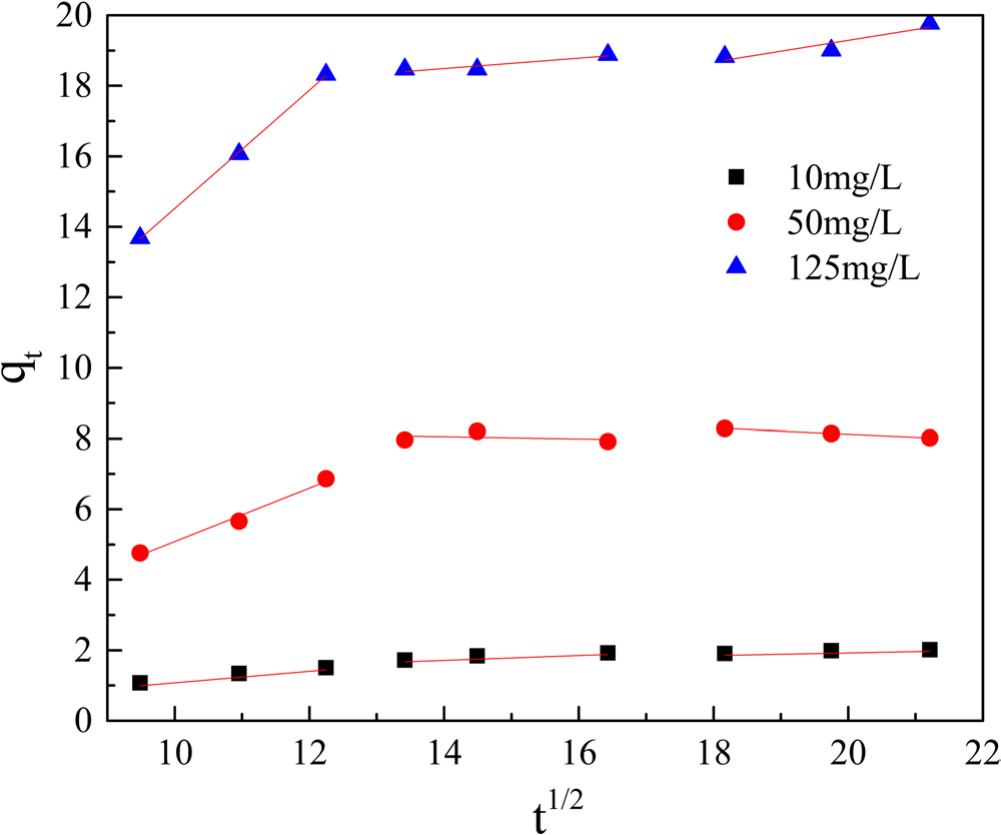
Intra-particle diffusion model.

For the *pseudo*-first order, the *pseudo*-second order and the intra-particle diffusion models, ln(*q*_e_-*q*_t_) to time(t), t/*q*_t_ to time and q_t_ to t^1/2^ were plotted (Figs 9–12), and liner fit derived.

As is shown in Fig. 9, the proper contact time of GAD beads with Mn(II) is crucial to favor adsorption performance. At the beginning of reaction, the adsorption capacity was enhanced and the adsorption rate was fast with the times increased at 125 mg/L of Mn(II). After 210 min, the adsorption capacity slightly changed and gradually reached equilibrium. The initial concentration of Mn(II) in aqueous solution also influences the adsorption capacity: higher Mn(II) initial concentration relate with higher adsorption capacity. At 125 mg/L initial Mn(II) concentration, the adsorption capacity of GAD hydrogel beads is saturated at 19.81 mg/g while 2.07 mg/g when the initial concentration of Mn(II) is 10 mg/L. With the increase of initial concentration, the probability of GAD hydrogel beads contacting with Mn(II) in aqueous solution become higher, consequently the adsorption rate and the saturation capacity also increase^10^.

As shown in Figs 10 and 11 and Table 2, the adsorption data fit both the *pseudo*-first order and the *pseudo*-second order adsorption model, but *pseudo*-second order model was preferable. At initial concentrations of 10 mg/L, 50 mg/L and 125 mg/L, the correlation coefficients(R^2^) were above 0.97. Multi steps often are involved into adsorption: transport of solute molecules from the aqueous phase to the surface of the solid adsorbent, then diffusion of the solute molecules into the interior of the pores. Therefore, the intra-particle diffusion model was used to further describe the process of adsorption. The plot of q_t_ against t_1/2_ in Fig. 12 show a multi-linearity correlation, which may indicate the process could be divided into three stages (shown with linear sections). The first steeper slope represented the transport of Mn(II) ions from the aqueous solution to the adsorbent external surface through diffusion. The second slope was the diffusion of the Mn(II) ions into the pores of the adsorbent, which is rate determining, therefore to be a slow stage. The third slope was the equilibrium process, where the Mn(II) ions are adsorbed on the adsorption sites on the internal surface of the pores and the intra-particle diffusion begins to slow down because the solute concentration gets lower in bulk solution^50^.

**Table 2:** Parameters for adsorption kinetic study.

The initial concentration of Mn(II)(mg/L)		10	50	125
q_exp_(mg/g)		2.07	8.47	19.81
*Pseudo*-first order	q_cal_(mg/g)	1.59	3.11	11.23
	k_1_	0.0075	0.0062	0.0097
	R^2^	0.9476	0.5323	0.7396
*Pseudo*-second order	q_cal_(mg/g)	2.49	9.50	21.09
	k_2_	0.0041	0.0017	0.0014
	R^2^	0.9866	0.9733	0.9954
Intra-particle diffusion	k_i1_	0.1565	0.7575	1.6744
	k_i2_	0.0665	−0.0320	0.1471
	k_i3_	0.0361	−0.0881	0.3080
	R_1_^2^	0.9908	0.9857	0.9996
	R_2_^2^	0.9334	0.1019	0.8763
	R_2_^3^	0.9443	0.9995	0.8720

#### Adsorption Thermodynamics

Initial Mn(II) concentrations 10–150 mg/L were prepared and reacted with adsorbents in an incubator shaker (150 rpm) for 120 min at pH 9, at the temperatures of 298 K, 308 K and 318 K individually. The dosage of GAD was 150 mg/50 ml. The Langmuir, Freundlich and Temkin isotherm models were applied to fit the experiment data collected. These are described below.

6 $$\frac{{C}_{e}}{{q}_{e}}=\frac{{C}_{e}}{{q}_{{\rm{\max }}}}+\frac{1}{{q}_{{\rm{\max }}}{K}_{L}}$$

7 $$\mathrm{ln}\,{q}_{e}=\,\mathrm{ln}\,{K}_{F}+\frac{1}{n}\,\mathrm{ln}\,{{C}}_{e}$$

8 $${q}_{e}=B\,\mathrm{ln}\,A+B\,\mathrm{ln}\,{C}_{e}$$

where the C_e_ is the mass concentration of Mn(II) when the system is at equilibrium; K_L_ is the Langmuir adsorption constant. K_F_ is a Freundlich constant related to adsorption capacity and 1/n is an empirical parameter giving an indication of the favorability of adsorption^51^. It is assumed that when 1/n is larger than 2.0 it is difficult to adsorb; A is the Temkin adsorption isotherm binding constant and B is the constant related to heat of adsorption.

**Figure 13:**
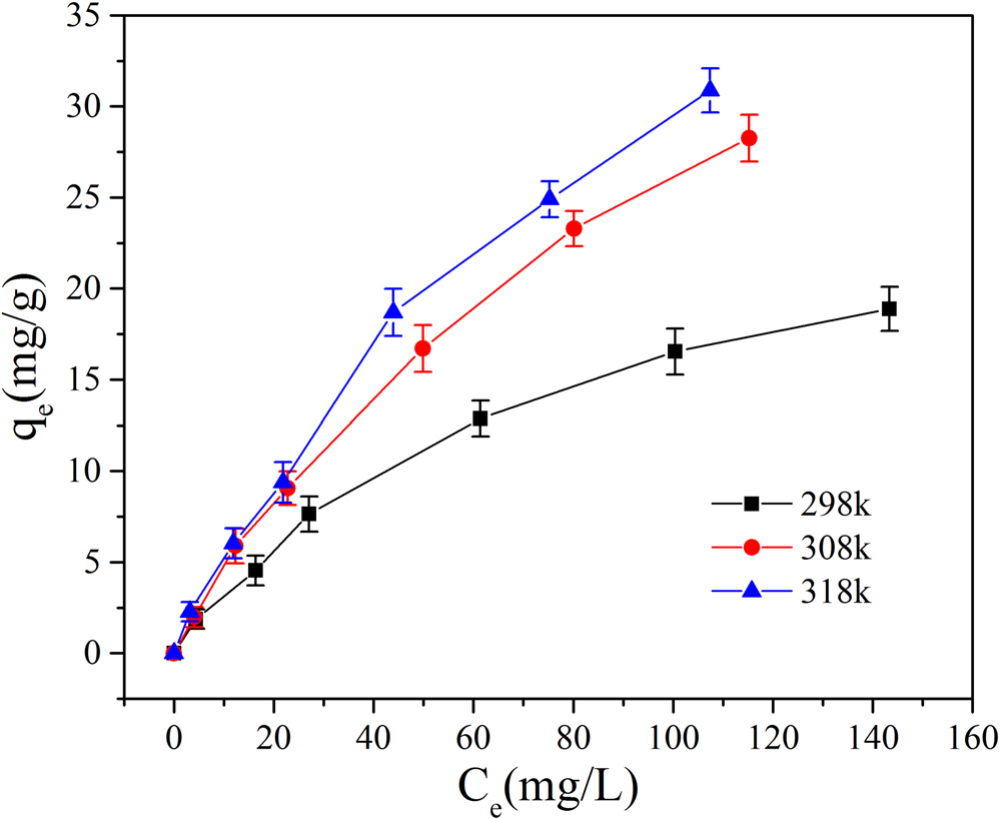
Effects of temperature on removal of Mn(II) by GAD.

As shown in Fig. 13 the adsorption capacity of GAD hydrogel beads increases when the temperature and the initial concentration of Mn(II) increase, indicating that the adsorption is endothermic. The higher temperature accelerates the activity of Mn(II) ions and the higher initial concentration of Mn(II) increases the probability of reaction of Mn(II) ions in the solution, therefore driving the reaction forward.

**Figure 14:**
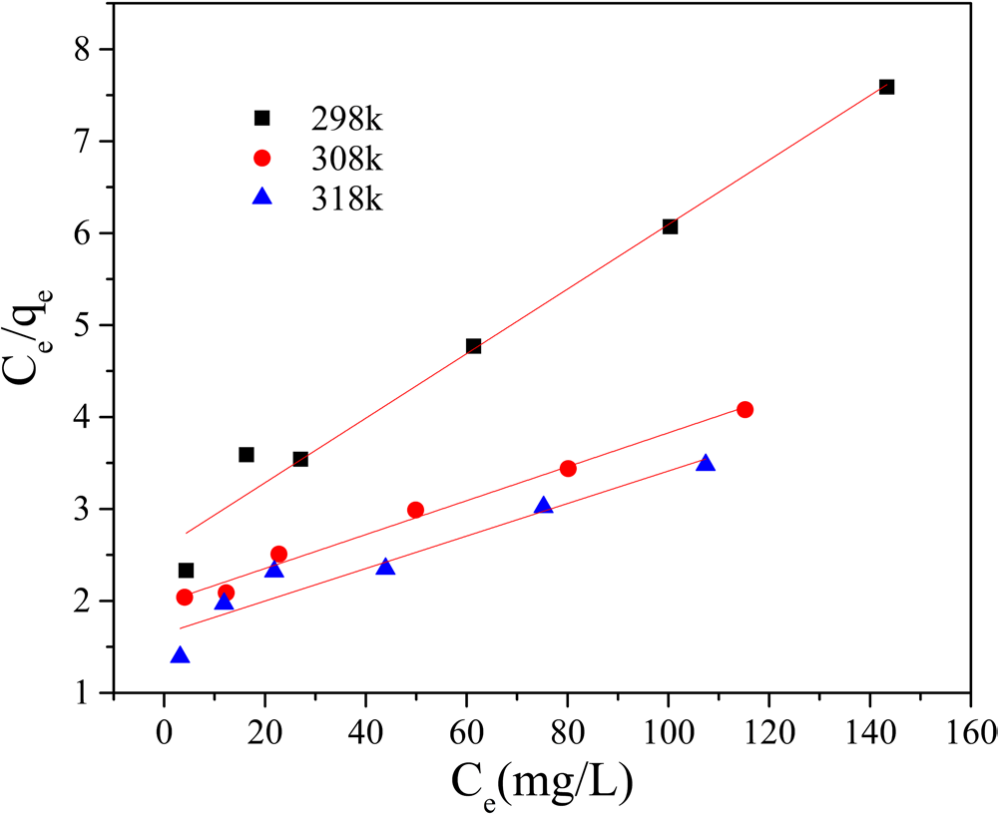
The Langmuir adsorption isotherm.

**Figure 15:**
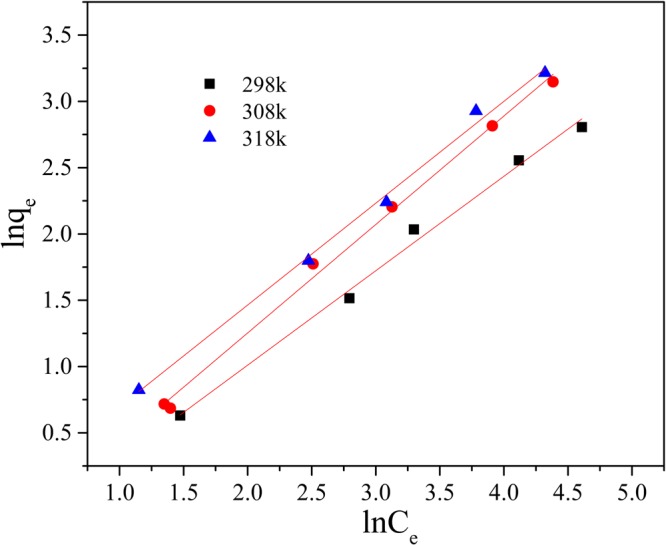
The Freundlich adsorption isotherm.

**Figure 16:**
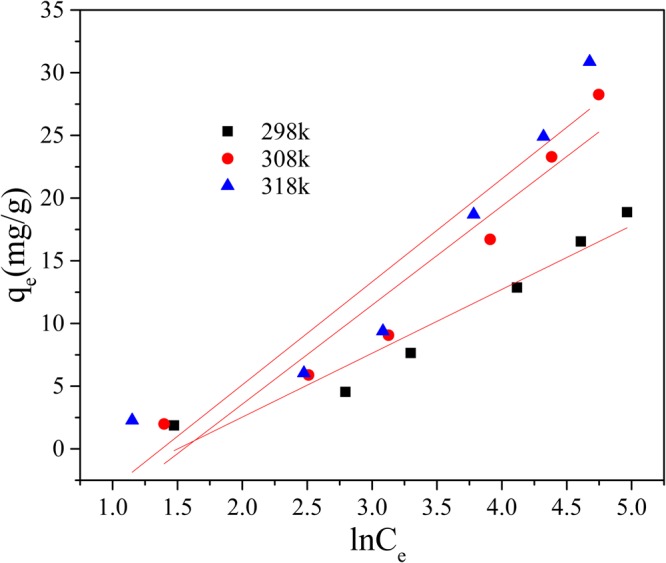
The Temkin adsorption isotherm.

As shown in Figs 14 and 15 and Table 3, both the Langmuir and Freundlich isotherms give a good fit to the experimental data (R^2^ > 0.92 at least) but not Temkin adsorption modle (R^2^ of 0.94 in maximum in Fig. 16). According to the Langmuir isotherm equation, the maximum adsorption capacity of the GAD hydrogel beads reaches 56.49 mg/g. The correlation coefficients of the Freundlich, listed in Table 3, are all above 0.99 under different temperature, implying better fitting than the Langmuir isotherm. The Freundlich constant 1/n indicate the sorption intensity of the sorbent. It is empirically accepted adsorption is wonderful when 1/n ⊆ (0.1, 0.5); it is easy to adsorb when 1/n ⊆ (0.5, 0.1); it is difficult to adsorb when 1/n > 1^52^. As shown in Table 3, the 1/n is 0.68 at 298 K; 0.79 at 308 K; 0.76 at 303 K. The 1/n value of GAD hydrogel range from 0.5 to 0.8, implying that Mn(II) ions can be effectively adsorbed by GAD hydrogel beads^53^.

**Table 3:** Parameters of isotherm model.

T(K)	Langmuir			Freundlich			Temkin		
	q_max_ (mg/g)	K_L_	R^2^	K_F_	1/n	R^2^	A	B	R^2^
298	28.49	0.0135	0.9804	0.7204	0.6795	0.9902	0.8606	5.0914	0.9447
308	54.34	0.0093	0.9891	0.7319	0.7892	0.9929	0.2119	7.8984	0.9283
318	56.49	0.0108	0.9248	0.9516	0.7599	0.9960	0.2521	8.2133	0.9007

The adsorption capacity of Mn(II) with various adsorbents were summarized in Table 4, including inorganic porous material with immense surface area, natural and synthetic polymer, demonstrating the GAD hydrogel beads prepared in this study perform better than others. For instance, the adsorption capacity of GAD beads increase 27 folds comparing to commonly used granular activated carbon, four times increase than (PVA/CS) hydrogel and double the Al-zeolite composites’ capacity. It is fair to point out that direct comparison these adsorbents maybe inaccurate because of the characteristics and experimental conditions differences of adsorbents. However, it remains appreciate that GAD beads have great potential in treat actual wastewater than other previously reported adsorbents.

**Table 4:** Comparison of adsorption capacity of Mn(II) onto various adsorbents.

Adsorbent	Adsorption capacity (mg g^−1^)	pH	References
Granular activated carbon	2	7	^56^
Surfactant modified alumina	2.04	4.04–8.05	^11^
Palm fruit bunch	2.21	N/A	^59^
Amberlite IR-120H	4.9	7	^56^
Natural zeolite	7.68	6	^60^
Polyvinyl alcohol/chitosan (PVA/CS) hydrogel	10.515	5	^10^
Activated carbon from bean pods waste	23.4	5–6	^61^
Al-zeolite	25.12	6	^60^
Mesoporous carbons	40	7	^62^
Graphene oxide/Sodium alginate beads	56.49	6	This study

Thermodynamic parameters was calculated by equations shown below^54^:

9 $${\rm{\Delta }}G=-\,RT\,\mathrm{ln}\,{K}_{D}$$

10 $${\rm{\Delta }}G={\rm{\Delta }}H-T{\rm{\Delta }}S$$

11 $$\mathrm{ln}\,{K}_{D}=\frac{{\rm{\Delta }}S}{R}-\frac{{\rm{\Delta }}H}{RT}$$

where the K_D_ is the thermodynamic equilibrium constant^55^; ΔG is the free energy of adsorption, kJ/mol; ΔH is the enthalpy of adsorption, kJ/mol; ΔS is the entropy of adsorption, J/(mol·K), R is the ideal gas constant, 8.314 J/(mol·K), T is the temperature in adsorption.

Based on the date of the experiment that the removal of Mn(II) by GAD hydrogel beads, the K_D_ was obtained by plotted ln(q_e_/C_e_) versus q_e_ and extrapolated to zero q_e_. Then, graphed lnK_D_ vs 1/T, ΔH and ΔS were calculated by the formula (11) and the ΔG can be calculated by formula (10).

**Figure 17:**
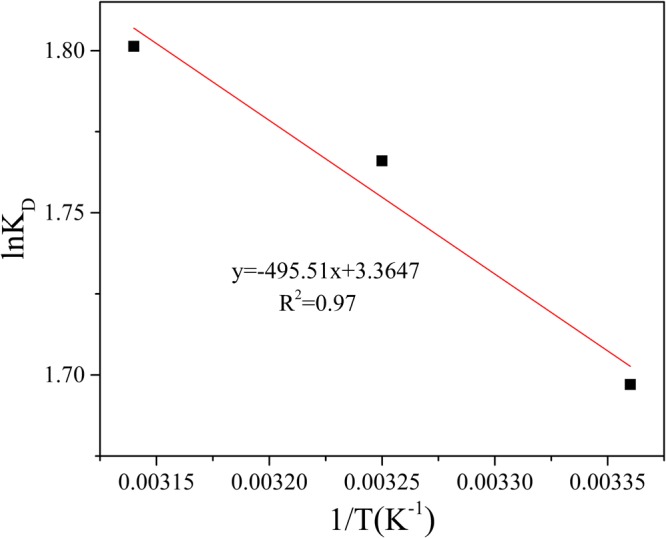
Linear fit for the Thermodynamic parameters for Mn(II) sorption onto GAD hydrogel beads.

Like any other spontaneous process, a favorable adsorption occurs only when it is associated with a negative free energy change(ΔG). As shown in Table 5, the values of ΔG for adsorption of Mn(II) ions were −4.22, −4.50 and −4.78 kJ/mol at 298, 308 and 318 K. The negative ΔG at all studied temperatures infer that the adsorption of Mn(II) on GAD would follow a spontaneous pattern. The ΔG value decreased with an increase in the temperature from 298 K to 318 K, revealing an increase in adsorption of Mn(II) with increasing temperature. From Fig. 17, ΔH and ΔS values were calculated as 4.12 kJ mol^−1^ and 0.028 kJ mol^−1^ K^−1^, individually. The positive value of ΔH and ΔS suggested the endothermic nature of the adsorption process^56^ and increased randomness at the solid-solution interface^57^.

**Table 5:** Thermodynamic parameters for Mn(II) sorption onto GAD hydrogel beads.

T(K)	△G (kJ/mol)	△H (kJ/mol)	△S (kJ/(mol•K)
298	−4.22		
308	−4.50	4.12	0.028
318	−4.78

#### Reusability Studies

**Figure 18:**
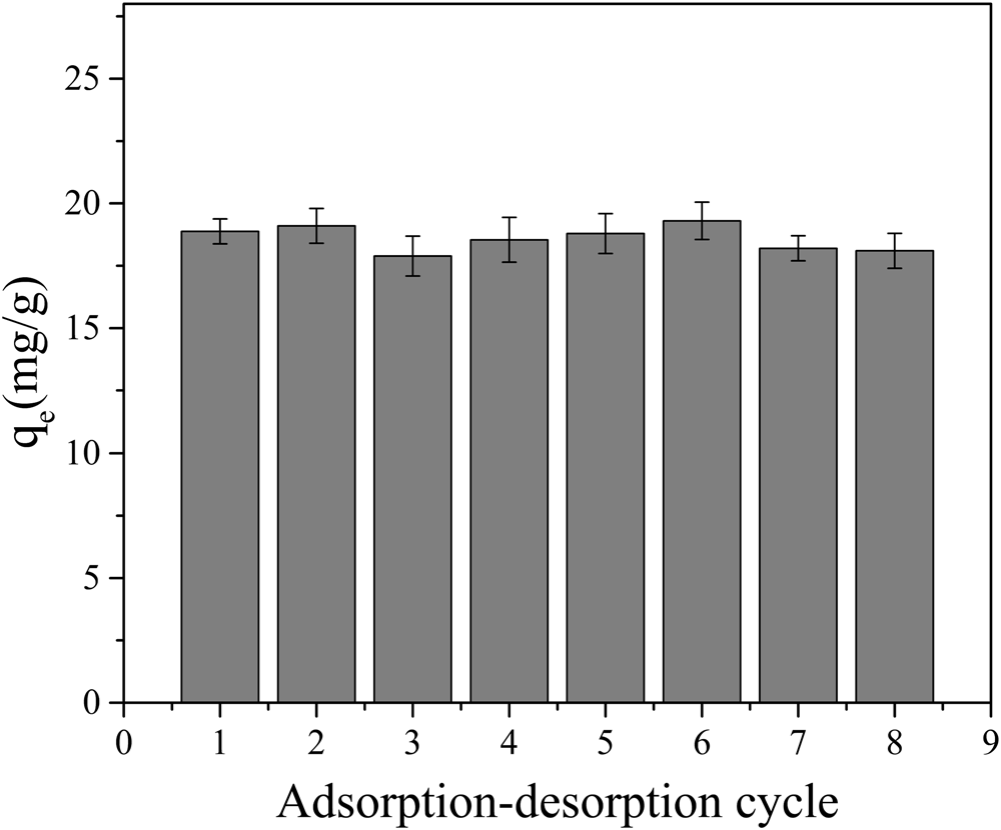
Adsorption-desorption cycle of GAD.

Since regeneration and reusability are important factors of an adsorbent, plus the high cost of GO based material^58^, the reusability of GAD hydrogel beads in current study need to considered as well. In this experiment, HCl was chosen as desorbent, the adsorbed Mn(II) can be elute with the treatment of 0.1 mol/l HCl. When HCl was used as an eluent, the interaction between GAD and Mn(II) was disrupted, and subsequently the Mn(II) was released into the eluent. In order to investigate the reusability of GAD, the adsorption-desorption was repeated seven times using the same sample adsorbent. As the spent adsorbent was recovered, the acquired GAD was reused for the adsorption of Mn(II) and the results were shown in Fig. 18. During seven consecutive cycles of adsorption-desorption, the slight diversity adsorption capacity of GAD hydrogel was observed by almost remained unchanged at 18.11 mg/g. The results clearly showed that GAD hydrogel in question could be reused without significantly losing its adsorption ability.

## Conclusions

In the adsorption of Mn(II) from aqueous solution by GAD hydrogel beads at the pH of 6 and dose of 150 mg, the removal efficiency was the optimum. In the adsorption process, kinetic analysis shows a good fit for a pseudo-second order kinetic model. The Freundlich isotherm model was the best fit for the adsorption of Mn(II) by GAD hydrogel beads, and the theoretical capacity of adsorption was found to be 56.49 mg/g was higher than other similar sorbents. The thermodynamic analysis showed the adsorption of Mn(II) was spontaneous, endothermic and entropy favorable. After seven time of adsorption-desorption cycle, the adsorption capacity of GAD hydrogel remained unchanged at 18.11 mg/g. Based on abovementioned results and observation we believe that the GAD hydrogel beads fabricated and protocol developed in this work can be expanded to other pollutants containing wastewater treatment, such as cationic Sb2+ which is undergoing clearance trial.

## Electronic supplementary material

Supplementary Information
