# Supplementary material for: Removal of Mn (II) by Sodium Alginate/Graphene Oxide Composite Double-Network Hydrogel Beads from Aqueous Solutions
Source: Sci Rep. 2018 Jul 16;8:10717. doi: 10.1038/s41598-018-29133-y (PMC6048064; doi:10.1038/s41598-018-29133-y)
Supplement: Supplementary file 1 — Supplementary Information [file 41598_2018_29133_MOESM1_ESM.pdf]

Removal of Mn (II) by Sodium Alginate/Graphene Oxide Composite Double-Network Hydrogel Beads from Aqueous Solutions

Xiuzhen Yang[1], Tengzhi Zhou[1], Bozhi Ren[1], Andrew Hursthouse[1,2], Yuezhou Zhang[3*]

[1] College of Civil Engineering, Hunan University of Science and Technology, Xiangtan 411201, China yxzhy1102@126.com; zhoutenzhi123@163.com(T.Z.); renbozhi@126.com(B.R.)

[2] School of Science & Sport, University of the West of Scotland, Paisley PA1 2BE, UK; andrew.hursthouse@uws.ac.uk

[3] Pharmaceutical Sciences Laboratory, Faculty of Sciences and Engineering, Åbo Akademi University, FI-20520, Turku, Finland

* Correspondence: e-mail: zyuezhou@126.com; Tel.: +86-18073165540

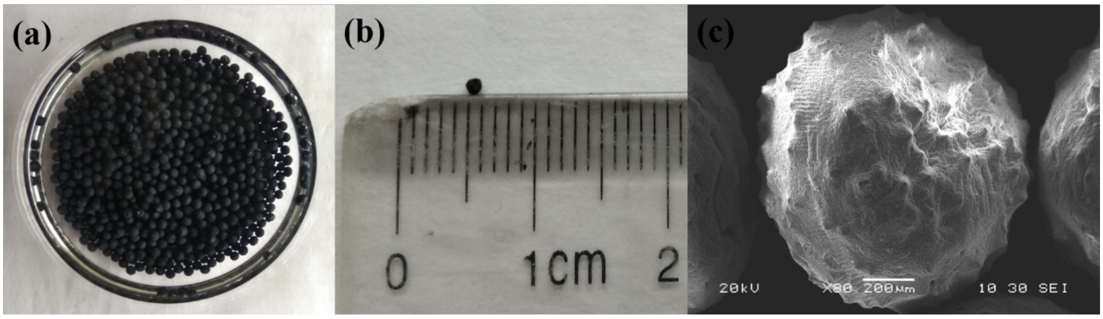

Fig.S1 The overview and individual structural feature of GAD

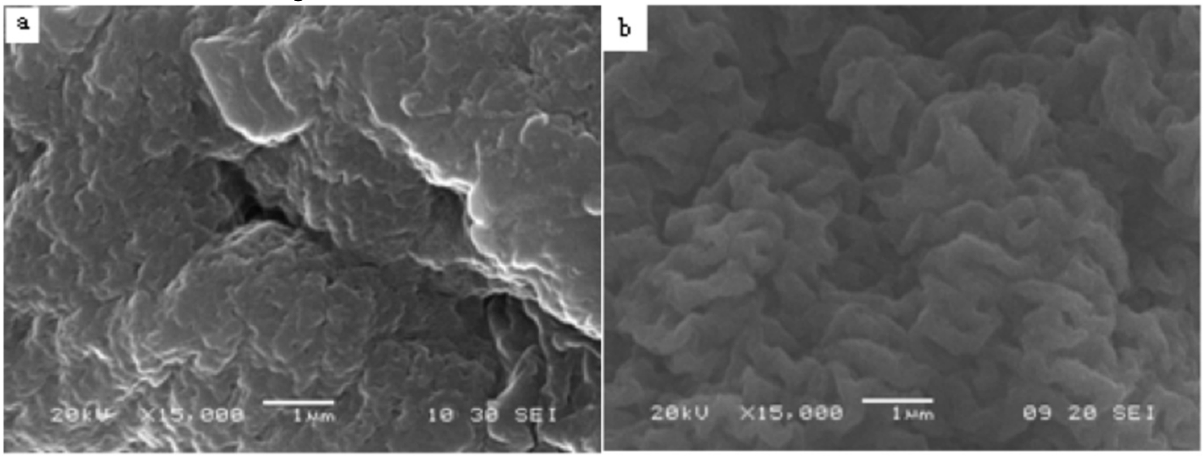

Fig.S2 SEM of a) the typical morphology of single network of Sodium Alginate and b) the morphology of Sodium Alginate/Graphene Oxide composite in this study
